# Response to “Development and Validation of the User Version of the Mobile Application Rating Scale (uMARS)”

**DOI:** 10.2196/mhealth.6419

**Published:** 2017-06-09

**Authors:** Shaira Baptista, Brian Oldenburg, Adrienne O'Neil

**Affiliations:** ^1^ Centre for Health Equity Melbourne School of Population and Global Health The University of Melbourne Melbourne Australia; ^2^ Centre for Mental Health Melbourne School of Population and Global Health The University of Melbourne Carlton Australia

**Keywords:** mobile apps, mhealth, app quality

We read with interest the article, “Development and Validation of the User Version of the Mobile Application Rating Scale (uMARS)," by Dr Stoyanov et al [1]. The authors report on the development and validation of the user version of the Mobile Application Rating Scale (uMARS). If applied appropriately, this scale has the potential to improve the quality and standardization of reporting, and assist in the progress of the scientific evidence base in mHealth research. The uMARS is based on the original ‘expert’ Mobile Application Rating scale (MARS), which consists of items identified from a literature search of web and mobile application (app) quality rating criteria, and that were tested for reliability using 60 wellbeing apps from the iTunes store [2]. The authors report on how the MARS was adapted into the uMARS for lay users by simplifying items and removing those that require professional or content expertise. Both scales are similar in structure, and include objective quality subscales: engagement, functionality, aesthetics and information quality, as well as a subjective quality subscale.

A reliable measure of app quality for end-users is urgently needed as health apps continue to proliferate without rigorous evaluation [3]. Although there have been notable attempts to measure the quality of mHealth apps, there is as yet no widely accepted standardized method for end-users [4-6]. This limits the ability to identify and scale-up successful apps, thereby limiting the population impact of mHealth [3,7]. 

While there is no doubt that the development of the MARS and uMARS represent significant progress towards greater consistency and transparency in mHealth, we would argue that it would be further strengthened by both a clear conceptual definition of app quality as well as a theoretical framework for testing app quality. To that end, we propose considering how the perception of app quality differs between experts (health professionals, researchers, app developers) and lay/end-users. For example, a health professional assesses the quality of a health app in order to identify apps to recommend to their patients. On the other hand, an end-user will assess the quality of a health app with the intent to use it. Indeed, evidence suggests that health professionals consider clinical effectiveness of mHealth apps to be most important, whereas consumers are looking for a seamless user experience, reduced data entry burden, and integration with their health care experience [8,9]. An important implication of these differences is that the simple adaptation of ‘expert’ items of the MARS may not accurately and adequately reflect the ‘end-user’ perspective on app quality. More research is needed to understand how expert and end-user perspectives on app quality differ in terms of their expectations, needs, preferences and attitudes. 

Secondly, the authors have used current literature to inform the development of the MARS and the uMARS. However, because the field is in its infancy, the development of an mHealth quality scale cannot rely solely on this inductive approach as there is much we do not know about how mHealth apps are accessed and evaluated by users. We suggest that the authors examine the substantial body of theoretical research on usability and engagement with technology to improve the comprehensiveness of the uMARS. 

We also recommend more research on how the perception of quality varies with respect to these technologies, especially from the user perspective. For example, there are clear distinctions between the functionality, programmability, interactivity and features of internet-based interventions and mobile apps. While web-based interventions imply that a user accesses information via the Internet from any connected device, mHealth involves a complex relationship between a user and their portable, personal mobile device [10]. Additionally, mobile apps offer greater functionality, personalisation and real-time interactivity. Thus, although there are many similarities between web and mobile app interventions, more research is needed to inform how effectively items describing the quality of a web based intervention translate into items measuring mobile app quality. 

Finally, the MARS and uMARS are relatively new advances in mHealth, and to date the MARS has been applied to the review of a handful of apps, including mindfulness apps [11], heart failure symptom monitoring apps, self-care management apps [12], and weight management apps [13]. Although there is an urgent need for consistent and transparent evaluation and reporting of app quality, especially from the user perspective, much more research is needed to validate the uMARS before it can be widely adopted and included into standardised mHealth reporting guidelines such as mERA and CONSORT-EHEALTH [14,15]. Further reducing the length and complexity of the response options of the uMARS will make it easier for researchers and others to apply the scale in their research. 
